# Autophagy, Mitophagy and MicroRNA Expression in Chronic Hepatitis C and Autoimmune Hepatitis

**DOI:** 10.1007/s12253-020-00799-y

**Published:** 2020-03-02

**Authors:** Tímea Szekerczés, Alíz Gógl, Ildikó Illyés, József Mandl, Katalin Borka, András Kiss, Zsuzsa Schaff, Gábor Lendvai, Klára Werling

**Affiliations:** 1 2nd Department of Pathology, Semmelweis University, Üllői 93, 1091 Budapest, Hungary; 2 Department of Medical Chemistry, Molecular Biology and Pathobiochemistry, Semmelweis University, 1094 Budapest, Hungary; 3 2nd Department of Internal Medicine, Semmelweis University, 1088 Budapest, Hungary

**Keywords:** Chronic hepatitis, autophagy, mitophagy, microRNA, immunohistochemistry

## Abstract

Although the role of autophagy has been implicated in several forms of chronic hepatitis, it is still not fully understood. Active autophagy eliminates damaged molecules and organelles (such as mitochondria) by lysosomal degradation. In the present study, we aimed to examine and compare autophagy activity in chronic hepatitis C (CHC) and autoimmune hepatitis (AIH) by detecting the expression of autophagy (LC3 and p62) and mitochondrium-related (TOMM20) proteins, as well as the levels of selected microRNAs (miR-101, -155, -204 and − 224) known to be involved in the regulation of autophagy. In addition, the expression levels were related to pathohistological parameters. Liver biopsy samples, including 45 CHC and 18 AIH cases, were immunohistochemically stained for LC3, p62 and TOMM20 and the expression of miRNAs was determined using real-time PCR. We found elevated LC3 and p62 in AIH samples as compared with CHC ones, indicating an activated autophagy that is impaired in AIH as no degradation of p62 seemed to occur. Moreover, p62 showed strong correlation with necroinflammatory grades in the AIH group. The observed elevated levels of TOMM20 and p62 suggest a less efficient elimination of damaged mitochondria in AIH as opposed to CHC, in which autophagy seems to have a more active function. The level of miR-101 was increased in case of CHC as compared with AIH, however, miR-155, -204 and 224 resulted in no expressional. Furthermore, miR-224 level correlated with steatosis and miR-155 expression with fibrosis stage in CHC. In conclusion, dissimilar autophagic activity was observed in CHC and AIH, suggesting a close association between impaired autophagy and severity of necroinflammation. This impairment may not be regulated by the analyzed miRNAs. Nevertheless, miR-224 and − 155 seem to be associated with CHC progression.

## Introduction

Hepatitis C virus (HCV) infection is one of the main health problems affecting 71 million people worldwide [1]. Despite the remarkable healing effect of the newly introduced direct-acting antivirals (DAAs), several issues are still not resolved or are under dispute [2, 3]. One of these is the role autophagy may play in the pathogenesis of chronic hepatitis C (CHC), which has been in the focus of several recent studies [4–6].

Autophagy, this evolutionarily conserved cellular catabolic process, is responsible for the elimination of predominantly damaged molecules and organelles via delivering them to lysosomes for degradation. Cell growth, development and homeostasis are the major processes in which autophagy is involved in order to maintain the balance between synthesis, degradation, and subsequent recycling of cellular products [7]. Several stress factors, including viral infection and autoimmunity, are known to activate autophagy [4, 8, 9]. Selective mitochondrial autophagy, termed mitophagy is particularly important in the elimination of mitochondrial dysfunction arising from aging and damage, thus, mitophagy plays crucial role in the reduction of oxidative stress and maintenance of cellular homeostasis [4, 6, 10–12].

An activated autophagy process is described as being beneficial for the replication and life cycle of HCV, since it protects the persistence of the virus in multiple ways. As it is known, intracellular membraneous web-associated virus replication and production of structural and non-structural HCV proteins lead to endoplasmic reticulum (ER)-stress and unfolded protein response (UPR), by which HCV indirectly induces autophagy through the activation of the UPR-induced signaling pathway [8, 13, 14]. ER-stress causes mitochondrial injury and leads to mitophagy [13, 15] as demonstrated in HCV infection [10]. In addition, HCV-induced autophagy is capable of suppressing the innate immune response and thus interferon (IFN) production, too [3, 13]. All these maintain the persistence of HCV infection and the pathogenesis of CHC without causing direct cytopathic effect on hepatocytes [3].

Autoimmune hepatitis (AIH) is another form of chronic hepatitis with an unknown etiology. Activated immune cells, involving cytokines and macrophages, characterize the disease [16], however, overall knowledge about autophagy in AIH is limited. Autophagy is known to be involved in the activation of adaptive immunity, promoting the direct elimination of pathogens and simultaneous antigen processing and presentation to T cells [17]. Autophagy is therefore believed to be linked with the immune response seen in AIH, with involvement of several autophagy-related genes and various pathways via mechanisms that are still unresolved [9].

The process of autophagy involves formation of phagophores, closure of autophagosomes and fusion of lysosomes with autophagosomes, in which the sequestrated cargo is digested by acidic hydrolases [8, 13]. Proteins found to be indicative of autophagy include LC3 (microtubule-associated protein light chain 3α) and p62 (or SQSTM1, sequestosome-1). LC3 becomes associated with the newly formed phagophore and interacts with p62, leading to the closure of the phagophored cargo (autophagosome) [8, 13]. TOMM20 is a protein in the outer mitochondrial membrane, responsible for recognizing and sorting precursor proteins for the biogenesis of mitochondria. Its expression is indicative of the available mitochondria in the cell, either damaged or active, and may correlate with mitophagy [18–20].

Several microRNAs (miRNAs) have been implicated in the regulation of autophagy by interfering with gene expression at post-transcriptional level. For example, miR-155 induces the formation of autophagophores via downregulating mTOR signaling but it impairs lysosomal degradation, as shown in alcoholic liver disease (ALD) [21]. miR-101 is thought to be a key regulator of the autophagy process and is a potent inhibitor of rapamycin-induced autophagy [22, 23]. miR-204 attenuates tumor autophagy in hepatocellular carcinoma (HCC) [24]. Additionally, autophagy itself may also regulate the expression of certain miRNAs, for example, the degradation of miR-224 in HCC [25]. Relationship between miRNAs and autophagy may be important in the pathogenesis of liver diseases [22].

In light of the above, our aim was to compare the expression of autophagy and mitochondrium-related proteins, and the levels of selected miRNAs in liver biopsy samples taken from patients with CHC and AIH. Moreover, we related the data to steatosis, fibrosis stage and necroinflammatory grade of the chronic hepatitis samples.

## Material and Methods

### Patient Characteristics

Liver biopsy samples from patients with CHC (45 cases) and AIH (18 cases) were taken for diagnostic purposes and analysed retrospectively before any treatment had been started. All samples derived from the archives of the 2nd Department of Pathology, Semmelweis University, Budapest with approval from the National Ethics Committee (45727-2/2013/EKU). The study was performed according to the Declaration of Helsinki. Patients were between 18 and 73 years of age at the time of biopsy with average age of 48.8 years for CHC and 43.4 years for AIH cases. The CHC group consisted of 31 males and 14 females, the AIH group comprised 5 males and 13 females. Chronic hepatitis cases of mixed etiology (alcohol consumption, hepatitis B virus (HBV) or other viral infections, metabolic liver diseases, advanced cirrhosis, tumors, and drug treatments) were excluded.

### Histology

Liver core biopsy samples taken with Menghini needle were fixed in formalin and embedded in paraffin (FFPE). Hematoxylin-eosin (H&E) stained sections of 3–4 µm thickness were analyzed to establish the diagnosis, necroinflammatory grade (according to the Ishak modification of the Knodell histology activity index [26]) and fibrosis stage (using the METAVIR scoring system [27]). Picrosyrius red staining was applied to highlight connective tissue fibers. Necroinflammatory grades were scored from 0 to 18 and fibrosis stages from F0 to F4. The degree of steatosis was assessed as percentage of lipid droplets-containing hepatocytes and scored 0 (under 5%), 1 (5–33%), 2 (34–66%) and 3 (above 66%) [28].

### Immunohistochemistry

Immunohistochemical reactions were carried out on 3-4-µm-thick FFPE sections with Ventana Benchmark XT automated immunohistochemical staining system (Ventana Medical Systems; Tucson, AZ) using HRP multimer based, biotin-free detection technique according to the protocol provided by the manufacturers. Briefly, antigen retrieval for 30 minutes at 95 °C was followed by incubation with specific primary antibodies for 30 minutes at 42 °C. The primary antibodies were directed against LC3 (polyclonal rabbit, NB-100-2331, Novus Biologicals, Abingdon UK, dilution 1:200), p62 (monoclonal mouse, ab56416; AbCam Cambridge UK, dilution 1:1000) and TOMM20 (monoclonal mouse, F-10 sc-17764, Santa Cruz Biotechnology Inc., Oregon USA, dilution 1:200). Indirect biotin streptavidin system (iVIEW DAB Detection Kit, Ventana) was used for visualization of reactions. For negative controls, the specific primary antibodies were omitted and replaced by the antibody diluent. Cervical carcinoma tissues served as positive controls for LC3 and TOMM20, and alcohol-induced HCC samples for p62 as recommended by the manufacturers of the antibodies.

Evaluation of the immunohistochemical reactions was performed using semiquantitative analysis. Each reaction was given a score according to the intensity of staining (0 – no, 1 – minimal, 2 – weak, 3 – moderate, 4 – intensive and 5 – very intensive), as well as the degree of cell staining [(0) < 6%, (1) 6–20%, (2) 21–40%, (3) 41–60%, (4) 61–80% and (5) > 81%]. Finally, the scores for intensity and percentage were summarized (IHC score).

### RNA Isolation

Total RNA was isolated from 5 µm thick FFPE sections using RNeasy FFPE Kit (QIAGEN, Venlo, The Netherlands) according to the instructions of the manufacturer with modifications for co-purification of miRNAs [29]. Traces of genomic DNA were eliminated by DNase digestion (Turbo DNA-free kit, Ambion by Life Technologies, Carlsbad, CA, USA). The concentration of RNA was measured using a NanoDrop 1000 spectrophotometer (Life Technologies of Thermo Fischer Scientific, Waltham, MA, USA).

### Reverse Transcription (RT) and Quantitative Real Time PCR (qPCR)

The list of the 6 selected miRNAs is presented in Table 1. along with the ID codes for the TaqMan MicroRNA Assays (Life Technologies brand of Thermo Fisher Scientific Inc.) used for measuring the expression of these miRNAs as instructed by the manufacturer. RT reaction was carried out using TaqMan MicroRNA Reverse Transcription Kit (Applied Biosystem brand by Thermo Fisher Scientific Inc.) in a total volume of 7.5 µl containing 20 ng RNA preparation. The qPCR was performed using TaqMan Universal Master Mix II no UNG (Applied Biosystem brand by Thermo Fisher Scientific Inc.) in a final volume of 7.5 µl containing 0.65 µl of the RT product. The amplification reaction was run in triplicates on a LightCycler 480 Instrument II (Roche Diagnostics, Indianapolis, IN). Relative expression was calculated by the 2^∆Cq^ (where ΔCq = Cq_Ref_-Cq_miR_) formula, using the average of miR-140 and RNU6B as the most stable reference determined by NormFinder application [30].

**Table 1:** List of selected microRNAs

Assay ID	miRNA
002099	hsa-miR-224-5p
002253	hsa-miR-101-3p
002623	hsa-miR-155-5p
000508	hsa-miR-204-5p
001187	hsa-miR-140-3p
001093	RNU6B

### Statistical Analysis

All figures and statistical analyses were created using GraphPad Prism (version 5.01; California, USA) and Statistic v.13 (Stat-Soft Inc.,Tulsa, OK, USA) software. A *p-*value of 0.05 was set as the threshold for statistical significance. The differences between CHC and AIH in relation to steatosis, fibrosis stage and necroinflammatory grade were examined by non-parametric Kruskal-Wallis analysis of variance and Mann-Whitney U test. Correlation of steatosis, fibrosis stage and necroinflammatory grade with IHC scores and miRNA expression was performed using non-parametric Spearman rank correlation test.

## Results

### Histology

The established pathohistological parameters for the samples are listed in Table 2. No steatosis was present in 12 (26.7%) samples with CHC and in 14 (77.8%) samples with AIH; whereas liver steatosis could be detected in 33 (73.3%) CHC cases and 4 (22.2%) AIH cases. Fibrosis stage was found to be low (F1-F2) in 31 (68.9%) CHC and 7 (38.9%) AIH cases, whereas high (F3-F4) in 14 (31.1%) CHC and 11 (61.1%) AIH cases. Finally, mild necroinflammation was diagnosed in 25 (55.6%) CHC cases, whereas 11 (61.1%) AIH samples showed severe necroinflammation.

**Table 2:** Pathohistology of biopsy samples: steatosis, fibrosis stages and necroinflammatory grades

		CHC (n = 45)		AIH (n = 18)	
*Average years of age*		48.88		43.4	
*Gender* (*M/F*)		31/14		5/13	
*Steatosis*					
negative (0 = under 5%)		12		14	
mild (1 = 5–33%)		14		2	
moderate (2 = 34–66%)		14		2	
high (3 = above 66%)		5		0	
*Fibrosis stage (F)*					
F1	(F1-F2)	18	(31)	3	(7)
F2		13		4	
F3	(F3-F4)	3	(14)	10	(11)
F4		11		1	
*Necroinflammatory grade*					
mild (under score 6)		25		4	
moderate (7–12)		18		3	
severe (13–18)		2		11	

CHC: chronic hepatitis C, AIH: autoimmune hepatitis, M: male, F: female

### Evaluation of Immunohistochemical Reactions

**Fig. 1:**
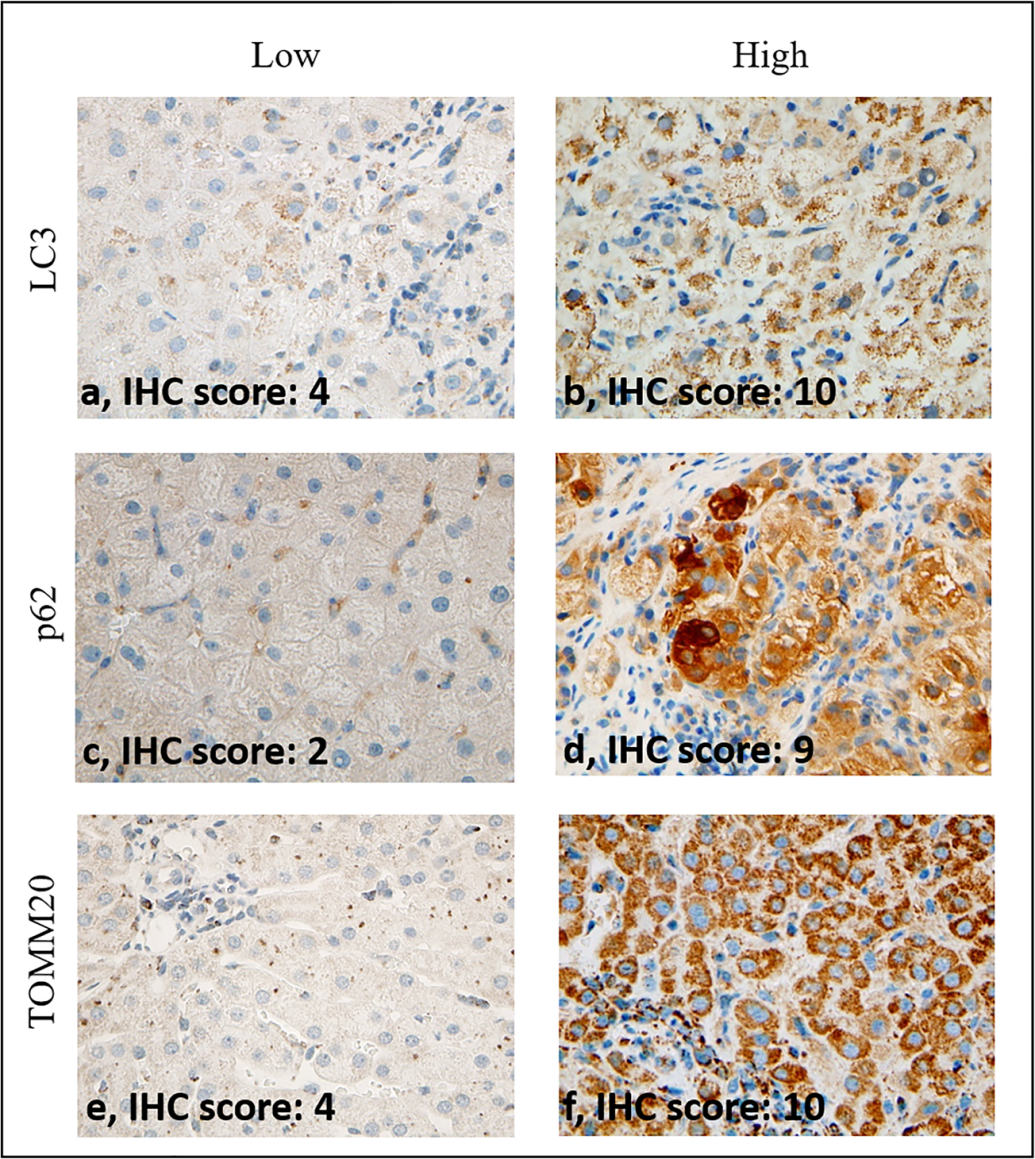
Representative images of low and high levels of LC3 (a, b), p62 (c, d) and TOMM20 (e, f) detected by immunohistochemistry (40x magnification).

LC3 and p62 stainings were rather diffuse, occasionally showing granular-focal cytoplasmic reaction with varying density both in hepatocytes and cholangiocytes, while TOMM20 exhibited brownish granular cytoplasmic reaction (Fig. 1). Altogether 42 CHC and 18 AIH samples provided evaluable immunohistochemical reaction for LC3, p62 and TOMM20.

**Fig. 2:**
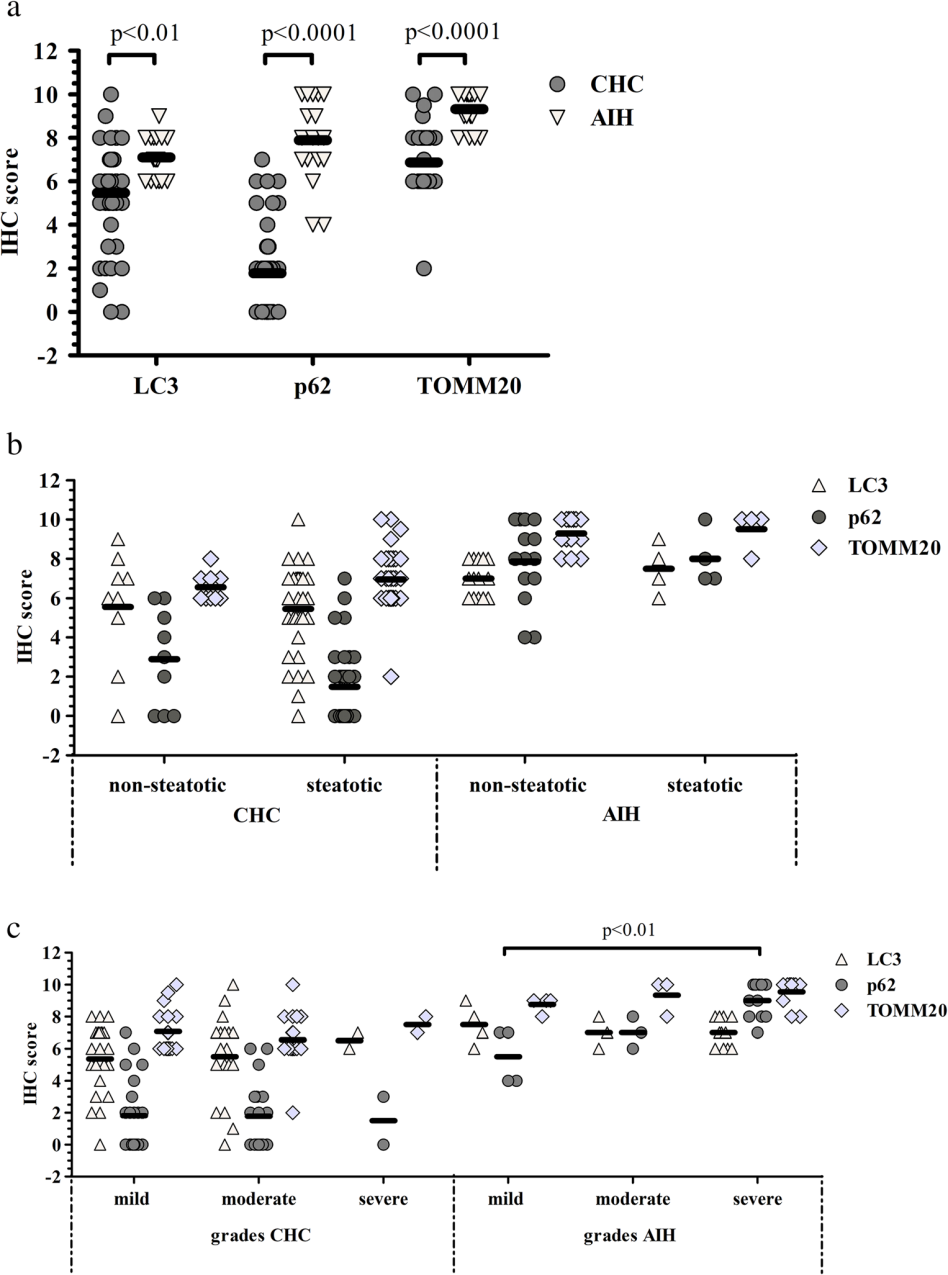
Semiquantitative evaluation of LC3, p62 and TOMM20 immunohistochemical reactions (a) and their association with steatosis (b) and necroinflammatory grade (c) in CHC and AIH.

Overall, AIH cases showed elevated LC3 (*p* < 0.01), p62 (*p* < 0.0001) and TOMM20 (*p* < 0.0001) levels as compared to CHC cases (Fig. 2a). When calculating ratios, the average LC3/TOMM20 ratios for the AIH and CHC groups were 0.76 and 0.80, respectively, while the average p62/TOMM20 ratio turned out to be 0.85 for the AIH and 0.26 for the CHC group.

No differences in the IHC scores of these proteins were observed when cases with or without steatosis were compared in either CHC or AIH samples (Fig. 2b). Nevertheless, decreased p62 could be found in the steatotic group of CHC samples as compared with the nonsteatotic group, however, the difference did not reach the set threshold for significance. Further, no association could be observed between fibrosis stages and LC3, p62, TOMM20 levels.

In regard to necroinflammatory grade, the level of p62 was significantly elevated (*p* < 0.0001) in severe grades of AIH as compared to mild grades (Fig. 2c). In addition, p62 showed strong correlation with necroinflammatory grade (r = 0.84; *p* < 0.0001) in AIH cases (Table 3) but no correlation was detectable in CHC samples (r=-0.03; *p* = 0.8445). Moreover, no association was observed between the levels of LC3, TOMM20 and necroinflammatory grade in relation to the two etiological groups.

**Table 3:** Correlations of p62 and miRNAs with pathohistological parameters

Sample group	Measured protein/miRNA	Pathohistological parameters	r	p
AIH	p62	Necroinflammatory grades	0.84	< 0.0001
CHC	miR-224	Steatosis	0.42	< 0.05
	miR-155	Fibrosis stage	0.56	< 0.01

CHC: chronic hepatitis C, AIH: autoimmune hepatitis

### miRNA Expression

**Fig. 3:**
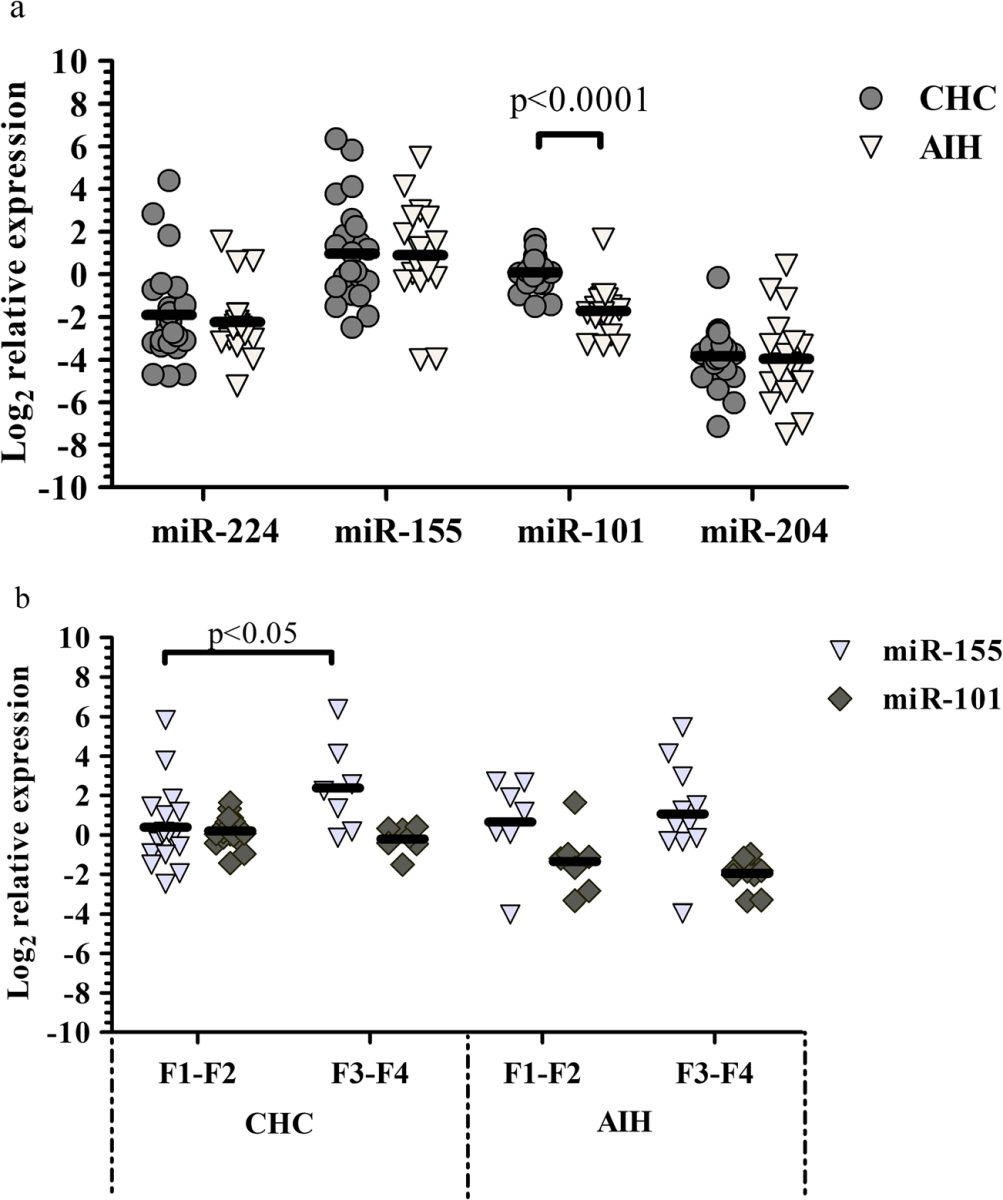
Relative miRNA expression (a) and the association of miR-155 and − 101 levels with fibrosis stages (b) in CHC and AIH

The levels of miRNAs could be determined in 24 CHC and 18 AIH samples. Expression of miR-224, -155 and − 204 was not statistically different between the CHC and AIH groups. On the contrary, an elevated level of miR-101 was observed in CHC as compared with AIH samples (*p* < 0.0001) (Fig. 3a).

No statistically different miRNA levels were found in either the CHC or AIH group when data were analyzed with regard to steatosis and non-steatosis cases. Nevertheless, the miR-224 level correlated with steatosis in CHC samples (r = 0.42; *p* < 0.05) (Table 3.) but not in AIH ones.

When miRNA expression was compared with the low (F1-F2), high (F3-F4) or separately with each (F1-F4) stage of fibrosis, no differences were found regarding miR-224, -101, -204 in either the CHC or AIH group or in case of miR-155 in the AIH group, but an elevated level of miR-155 (*p* < 0.05) was observed in the high as compared to the low stage of fibrosis in case of CHC (Fig. 3b). Furthermore, in the CHC group miR-155 level showed statistical difference between stages F1 and F4 (*p* < 0.05, data not shown) and miR-155 expression exhibited correlation with fibrosis stage as well (r = 0.56; *p* < 0.01) (Table 3.). Finally, no correlation was observable between miRNA expression and necroinflamatory grades in the CHC and AIH samples.

## Discussion

Autophagy acts as a bodyguard to protect cells, which involves concerted action to promote cell survival, for example in a state of stress when the cells are starved of nutrients or in case of a viral infection [5, 10]. The importance of autophagy has been emphasized in case of CHC, since it promotes HCV persistence and suppresses the activated immune response [15]. A possible link between autophagy and AIH has also been suggested [9], in which case however its role is not fully understood.

Upon examining LC3 and p62 as indirect indicators of autophagy function, the present study revealed increased levels of these proteins in AIH as compared with CHC. The level of LC3 *per se* indicates that autophagy is initiated, but may not reflect upon whether autophagy has proceeded or become impaired [21, 31]. A proceeding autophagy implies a low level of p62 since this protein becomes degraded together with the cargo in the autophago-lysosome [32]. Thus, increased LC3 and p62 in AIH suggest that autophagosome formation has occurred without an increase in lysosomal degradation, as has been demonstrated in case of p62 in a recent study [33]. Like further supported by Babuta and co-workers, increased levels of p62 and LC3 were found as the result of chronic alcohol intake in ALD, leading to disruption of autophagy function at lysosome level (impaired autophagic flux) [21].

Decreased level of p62 was observed in CHC contrary to AIH, suggestive of an autophagic function occurring at a much higher rate. This is supported by the observation that the level of p62 decreased within 3–6 days following HCV infection in Huh7.5 cells due to an increase in the autophagy response, which was indicated by an elevated level of LC3 [34].

Increased TOMM20 was detected in AIH as compared with CHC, which might suggest an increase in the number of active mitochondria and also the accumulation of damaged mitochondria not eliminated by autophago-lysosomes [18]. The latter is reported to imply an increased level of p62 in a nonalcoholic steatohepatitis (NASH) study [33]. Thus, the detected high TOMM20 and p62 might indicate that injured mitochondria are most likely not as effectively eliminated in AIH as in CHC.

According to the above mentioned NASH study, enhanced mitophagic level is characterized by elevated LC3/TOMM20 and decreased p62/TOMM20 ratios as the result of palmitic acid administered to mouse primary hepatocytes [33]. As regards the present study, the average LC3/TOMM20 ratios for AIH and CHC were found to be similar, but the average p62/TOMM20 ratio was three times higher in AIH than in CHC. This further signifies the presence of impaired mitophagy in AIH. The fact that Dynamin-related protein 1 (Drp1, a protein associated with mitochondrial dysfunction) was found to be colocalized with TOMM20 indicates that mitochondrial fission is involved in AIH, suggesting a link to hepatic necrosis [35]. It is intriguing that in the present study not only the expression of p62 increased but the necroinflammatory grades were also higher in AIH as compared with CHC, since we observed a strong correlation between p62 expression and necroinflammatory grade. This suggests that decreasing autophagic activity is linked to severity of inflammation; i.e. the more the cellular function becomes compromised due to an extensive inflammation, the less autophagy is capable of rescueing the cells, which may subsequently undergo necrosis. Dysregulated autophagy is reported to contribute to hepatic inflammatory injury and to enhance pro-inflammatory cytokine expression [36]. Stimulation of mTOR, a central issue in autoimmune disorders, also seems to contribute to inflammation in dysfunctional autophagy [37]. The fact that mTOR *per se* is an inhibitor of autophagy may explain the use of rapamycin as an mTOR inhibitor to treat AIH in the advanced stage [7, 9].

In CHC, the decreased p62/TOMM20 ratio along with the observed decreased level of p62 (one fourth of the expression observed in AIH) and reduced TOMM20 level (1.4 times less than in AIH) are indicative of a more active and proceeding autophagy process. This is supported by a study in which HCV was found to promote mitochondrial fission within 2 days following infection in Huh7 cells, where the damaged organelles were eliminated by mitophagy [38].

Altered lipid metabolism is linked to the life cycle of HCV considering that virions coated with lipoproteins facilitate hepatocyte entry via lipoprotein receptors, however, the HCV-induced ER-stress leads to steatosis [3]. Intracellular lipid droplets being in excess in the cells are degraded by an autophagy process called lipophagy [3–5, 22]. Vescovo et al. reported that autophagy acts against the alterations in lipid metabolism induced by HCV and steatosis may develop when autophagy is disrupted [39]. In the present study, no difference was observed in the expression of LC3 and p62 between steatotic or nonsteatotic chronic hepatitis samples (neither in CHC nor in AIH). Nevertheless, the level of p62 seemed to decrease in steatotic CHC as compared with nonsteatotic CHC, but the level did not reach the threshold for statistical significance.

Analysis of autophagy-associated miRNA expression may provide further knowledge about autophagic activity. Several miRNAs are known to downregulate autophagic activity (miR-101, -155 and 204) [21, 22] or their expression is known to be regulated by autophagy (miR-224) [25]. Autophagy-regulating miRNAs have been studied in HCV infection [40] but, to the best of our knowledge, no data is available on how these miRNAs are expressed in AIH.

In the present study, elevated miR-101 was found in CHC samples as compared with AIH cases, which raises concern as miR-101 is an autophagy inhibitor. Suppressed autophagy on the account of miR-101 is reported in breast cancer (targeting RAB5A, STMN1 and ATG4D), HCC (through EZH2) [22, 23] and in ischemic liver (via activating mTOR signaling pathway) [41]. Nevertheless, the levels of LC3 and p62 observed in the present study support the activation and not the inhibition of autophagy in CHC. Intriguingly, increased serum miR-101 expression has been found in HCV as compared with HCC [42]. Moreover, miR-101 has recently been reported to have an antifibrotic effect, most probably via downregulating the PI3K/Akt/mTOR pathway [43]. In the present study, association between miR-101 and fibrosis stage could not be observed in either CHC or AIH, however, when all of the CHC and AIH samples were combined into one group, miR-101 was found to correlate negatively with fibrosis stage (r=-0.36, *p* < 0.05) and necroinflammatory grade (r=-0.47, *p* < 0.01). It is to be taken into consideration, however, that the majority of CHC cases (68.9%) displayed low fibrosis stage, whereas the majority of AIH cases (61.1%) showed higher fibrosis stages. Additionally, higher miR-101 expression was found in CHC cases showing low fibrosis stage (F1, F2). Thus, we hypothesize that the relatively higher level of miR-101 might be enough for cells to avoid advanced fibrosis, but not enough to stop autophagy from happening. Further, miRNAs are known to exert their regulatory effect on a number of target genes, influencing several biological processes simultaneously; on the contrary, miR-101 may not be the only miRNA that simultaneously exerts an inhibitory effect on autophagy.

According to our findings, no association could be observed between miR-224 and autophagy. Nevertheless, the level of miR-224 correlated with steatosis in case of the CHC samples, but not in those of AIH. In a previous study, elevated level of miR-224 was found in steatotic CHC in comparison to nonsteatotic CHC [44]. Another study reported miR-224 to be elevated in the event of HCV recurrence in comparison to normal liver and also after administration of interferon/ribavirin treatment as compared with samples taken before treatment [45]. In addition, miR-224 is a predictive factor in the overall survival of hepatoblastoma [46]. miR-224 is a known oncomiR, described to promote proliferation in HCC. In this regard, the correlation of miR-224 with steatosis can be explained by the fact that steatotic CHC is one of the risk factors of HCC [13, 47].

In case of CHC, we found not only increased miR-155 expression in the higher fibrosis stages as compared with the lower stages, but also a correlation between miR-155 and fibrosis stage. In agreement with this, miR-155 knock-out mice were observed to be protected from early and advanced fibrosis in a CCl_4_ treatment model [48]. Regarding another regulatory aspect, miR-155 was not only found to initiate autophagy in ALD via downregulating mTOR, but also to impair the fusion of autophagosome with lysosome, leading to increased LC3 and p62 levels [21]. Our investigation is partly in agreement with this since high miR-155 expression (which was highest among the miRNAs) was observed in both CHC and AIH samples, but the levels of LC3 and p62 pointed to an impaired autophagy in case of AIH but not in CHC. Nevertheless, a marked increase in miR-155 expression was reported in HCV-infected patients, which seems to be involved in the regulation of antiviral immune response, promoting replication and persistence of HCV [49]. Further data on miR-155 in AIH is scarce, however, this miRNA may have a different role in inflammatory cell recruitment and liver damage [50].

As a summary, the different levels of LC3, p62, TOMM20 observed in the CHC and AIH cases indicate autophagy initiation in the examined samples of chronic hepatitis of different etiology. The increased p62 expression and its correlation with necroinflammatory grade observed in case of AIH suggest an autophagy process which is inhibited at the level of autophago-lysosomal degradation and is associated with extensive inflammation and overactivated immune response, leading to necrosis [51]. In relation to CHC, however, our findings provide further support for the evidence of a functioning autophagy process which seems beneficial for the persistence of HCV in hepatocytes and the pathogenesis of CHC so as to avoid a direct cytopathic effect on the host cells [3]. In our present study, we found no indication that the examined miRNAs had an inhibitory effect on autophagy in regard to either CHC or AIH cases. Instead, the miRNA levels correlated with steatosis (miR-224) and fibrosis (miR-155) in the CHC samples, which could possibly be associated with CHC progression.
